# Designing and Evaluating the Usability of a Machine Learning API for Rapid Prototyping Music Technology

**DOI:** 10.3389/frai.2020.00013

**Published:** 2020-04-03

**Authors:** Francisco Bernardo, Michael Zbyszyński, Mick Grierson, Rebecca Fiebrink

**Affiliations:** ^1^EMuTe Lab, School of Media, Film and Music, University of Sussex, Brighton, United Kingdom; ^2^EAVI, Department of Computing, Goldsmiths, University of London, London, United Kingdom; ^3^Creative Computing Institute, University of the Arts, London, United Kingdom

**Keywords:** application programming interfaces, cognitive dimensions, music technology, interactive machine learning, user-centered design

## Abstract

To better support creative software developers and music technologists' needs, and to empower them as machine learning users and innovators, the usability of and developer experience with machine learning tools must be considered and better understood. We review background research on the design and evaluation of application programming interfaces (APIs), with a focus on the domain of machine learning for music technology software development. We present the design rationale for the RAPID-MIX API, an easy-to-use API for rapid prototyping with interactive machine learning, and a usability evaluation study with software developers of music technology. A cognitive dimensions questionnaire was designed and delivered to a group of 12 participants who used the RAPID-MIX API in their software projects, including people who developed systems for personal use and professionals developing software products for music and creative technology companies. The results from questionnaire indicate that participants found the RAPID-MIX API a machine learning API which is easy to learn and use, fun, and good for rapid prototyping with interactive machine learning. Based on these findings, we present an analysis and characterization of the RAPID-MIX API based on the cognitive dimensions framework, and discuss its design trade-offs and usability issues. We use these insights and our design experience to provide design recommendations for ML APIs for rapid prototyping of music technology. We conclude with a summary of the main insights, a discussion of the merits and challenges of the application of the CDs framework to the evaluation of machine learning APIs, and directions to future work which our research deems valuable.

## 1. Introduction

Research on the design of music systems with artificial intelligence techniques goes back more than 30 years (Dannenberg, 1985). Much of this work has been motivated by the exploration and discovery of new sounds, music, and new forms of musicianship and performance (Miranda and Wanderley., 2008). Within this domain, research focused on the design of mapping strategies with interactive machine learning (IML)—i.e., using supervised machine learning (ML) for mapping between different kinds of inputs (e.g., sensor data, motion descriptors, audio features) and parameters of sound and music processes (e.g., Fiebrink et al., 2011; Françoise et al., 2013; Caramiaux et al., 2014)—has uncovered very meaningful advantages. They include, for instance, workflows with increased celerity and ease-of-use, intuitive exploration of complex mappings and high-dimensional parameter spaces, and increased utility of small training data sets. These findings are promising not only on their own, but also when considering the opportunities to broaden and accelerate innovation in music technology with ML (Bernardo et al., 2017). However, in order to facilitate the adoption of ML by music and creative software developers, the usability and the developer experience with new tools for designing, developing and using ML, must be considered and better understood.

IML approaches to building ML systems involve rapid cycles of human actions modifying an ML model and interactive examination of the outcomes of those modifications (Fails and Olsen, 2003). Unlike algorithm-driven approaches such as active learning, IML approaches entail human-driven cycles of model creation, change, and evaluation. As Amershi et al. (2014) write, IML enables “even users with little or no machine-learning expertise [to] steer machine-learning behaviors through low-cost trial and error or focused experimentation with inputs and outputs” (p. 106). IML approaches often provide ways for users to incorporate information about their goals or domain knowledge into the creation of an ML system, for instance by creating or curating training datasets that encode their understanding of the target behavior to be learned by an ML algorithm.

IML can be useful for ML problems in which the user's goal is to encode a human-understandable behavior into the system, and the user is capable of iteratively creating or curating training data to steer the model toward achieving the desired behavior. This is the case for many problems in music technology involving the analysis of audio, visual, and sensor data, in which a human user is capable of providing a supervised learning algorithm with examples of data paired with the desired labels. A musician could, for instance, pair examples of music clips, or segments of gestural data, with classifier labels indicating mood or instrumentation. Musicians and creative technologists have frequently used IML to support the creation of new sensor-based systems for embodied interaction and musical expression, such as the design of new musical instruments and new creative physical computing systems (Hartmann et al., 2007; Fiebrink et al., 2011; Katan et al., 2015).

In this paper, we present the design rationale and a usability evaluation of the RAPID-MIX API, a toolkit and application programming interface (API) for rapid prototyping music technology with interactive machine learning (IML). Our main objective is to explore how the design decisions and trade-offs of an API for rapid prototyping with IML affect its usability and the developer experience. We also identify specific design features in other ML APIs and toolkits and provide recommendations which our research and design experience suggests can be applied to other work. This work contributes a deeper understanding of ML API usability and its impact on the experience of music technologists and creative developers who are not ML experts and lack ML background. This work thus informs research and practice in the domains of API usability, human-centered ML, and music technology, where (to our knowledge) there is little research about human-centered design and evaluation of ML APIs.

The paper is structured as follows. This section introduces readers to concepts with background material on the design and evaluation of APIs, and contextualizes our work with information about other ML API and toolkit designs used in music technology. Section 2 describes the RAPID-MIX API as the main material and its underlying design assumptions. Section 3 describes the study with an adapted Cognitive Dimensions questionnaire. Section 4 presents a qualitative analysis of the results of the CDs questionnaire. In section 5, we discuss the main insights about the RAPID-MIX API design trade-offs and usability issues identified by the study. We also provide a set of recommendations for the design of ML APIs for prototyping music technology. We conclude in section 6 with a summary of the main insights and future work.

### 1.1. Design and Evaluation of APIs

Software developers integrating machine learning (ML) into their applications are likely to resort to third-party infrastructural software—that is, software that supports the development and operation of other software (e.g., middleware, software libraries and frameworks, online services, toolkits) (Edwards et al., 2003). The use of APIs—the developer-facing constituents of infrastructural software—is a standard and important practice in software engineering that prevails modularity and reusability (Fowler, 2004). Developers use API calls within their application code to extend their applications' capabilities with infrastructural software functionality.

APIs can provide potential savings in time and effort for common development tasks. However, developers making an informed decision about adopting an API may have to consider their previous experience with a specific API and API domain, as well as with the API conceptual model and the available design cues and patterns (Blackwell, 2002). Accessing the efficiency gains that APIs provide in relation to the cost of programming a custom solution is not straightforward though. Furthermore, the structure of the API and its documentation may have a significant impact on the API learning experience, given the ingrained assumptions about prior conceptual knowledge, target application scenarios, code examples, and learning resources provided (Robillard and Deline, 2011). When designing an ML API, the lack of consideration for these aspects can lead to a challenging and overwhelming learning experience.

Designing an API is a challenging task, let alone an ML API. An API must meet users' technical requirements—e.g., performance, robustness, correctness, stability, security (Henning, 2009). An API must be usable (Myers and Stylos, 2016) and provide effective learning (Robillard and Deline, 2011). An API must also be useful and provide an appropriate set of features for a space of potential client applications (Edwards et al., 2003). An ML API should provide the ability to train and evaluate existing ML algorithms on new data. A ML API can also incorporate a certain degree of domain expertise; for instance, by making available complete ML pipelines with predefined choices of algorithms and parameters (Mellis et al., 2017), pre-trained models for transfer learning (Jialin and Yang, 2010), or other functionalities that are likely to be useful for supporting application development in particular domains.

There exist different approaches to API design and evaluation. A designer-centric approach to API design is mostly based on the designer's taste or aesthetics^1^. This can be successful when the designer has an extensive experience in both API design and in the API application domain. Approaches based on API design heuristics use empirically-based knowledge that has been compiled into prescriptive guidelines or recommendations (e.g., Tulach, 2008; Cwalina and Abrams, 2009) There is, however, contradicting empirical evidence about the usability of certain API design heuristics (Myers and Stylos, 2016). User-centered design (UCD) approaches inform and drive API design with usability data (e.g., Clarke, 2010; Myers and Stylos, 2016). This can be useful to counteract misleading assumptions about API users who are not represented within the API designers' group. Nevertheless, usability approaches excessively focused on specific design features might fail to deliver in a more holistic way^1^.

Myers and Stylos (2016) provide a comprehensive review of different methods to measure and improve the design of APIs. One traditional approach is API peer reviews (Wiegers, 2002), where technical peers examine and give feedback. An alternative is the API Concepts framework (Scheller and Kühn, 2015), which automatically evaluates both the API and samples of client code, considering user characteristics (e.g., learning style, programming experience) among the critical factors of evaluation. Other methods have been adapted from traditional HCI and usability engineering, including empirical and task-specific evaluation techniques (e.g., think-aloud protocol, cognitive walkthrough) as well as heuristics-based techniques (Myers and Stylos, 2016).

Other approaches to API evaluation are based on the Cognitive Dimensions (CDs) of Notations framework (Green, 1989; Green and Petre, 1996). CDs are a “broad-brush” set of evaluation tools that support discussion about the design trade-offs of notations and information structures. The CDs have been previously to applied to the analysis and assessment of different types of music technology, including a music typesetting package (Blackwell and Green, 2000), music notation systems (Blackwell et al., 2000), sequencing interfaces (Nash, 2014), algorithmic composition software (Bellingham et al., 2014). API studies based on CDs typically either used the questionnaire originally developed by Blackwell and Green (2000), or a shorter or partially refactored version, specialized to a specific domain. For instance, the original CDs questionnaire Blackwell and Green (2000) was used by Austin (2005), who assessed the usability of a functional shading language for graphics programming. Diprose et al. (2017) used it to assess the abstraction level of an end-user robot programming API. Clarke and Becker (2003) derived a framework from the original CDs to characterize specifically how API design trade-offs met the expectations of the API users, and applied it to evaluate Microsoft ·NET class libraries. Watson (2014) applied Clarke's framework for improving API documentation planning. Wijayarathna et al. (2017) adapted Clarke's questionnaire for evaluating the usability of a cryptography API.

There is little research focusing on the human-centered design and evaluation of ML APIs. To our knowledge, there is no prior research which applies the Cognitive Dimensions framework in the evaluation of the usability of an ML API.

### 1.2. Machine Learning APIs and Toolkits for Music Technology

Developers are users of ML when they configure learning algorithms, and when they train, evaluate, and export models, or import the resulting pre-trained models into their music technology applications. When building IML or other “intelligent” systems for personal use in music performance and composition—i.e., end-user development (Lieberman et al., 2006)—or for others to use, in commercial applications of music technology, developers can employ custom-built learning algorithms. However, many developers will use general-purpose ML infrastructural software via API calls to build their applications, regardless of the specific end-user goal or end-user application usage.

Over the years, a number of general-purpose ML tools have been developed, including R packages such as Caret (Kuhn, 2008), graphical user interfaces (GUIs) such as Weka (Hall et al., 2009), and APIs such as scikit-learn (Buitinck et al., 2013). With the recent breakthroughs in deep learning, we observe an intensive push of ML development toolkits and APIs into the hands of developers—e.g., Google Tensorflow (Abadi et al., 2016), Apple CoreML^2^ and TuriCreate^3^, Pytorch^4^. While most of these APIs target ML experts, some of them cater to an audience of ML non-expert users. However, many of these APIs still remain difficult to use.

Other initiatives push for the democratization of ML using a domain-specific approach, i.e., within certain domains of application and more creative endeavors, which include the generation and control of media, such as image, video, and music. MnM (Bevilacqua et al., 2005) is a toolkit which allows users to create custom gesture-to-sound mappings using statistical methods such as principal components analysis^5^, hidden Markov models^6^ and other algorithms. This toolkit is implemented as a suite of externals for Max^7^, which is used extensively in the context of experimental music technology. These externals (i.e., processing components that are used within Max's graphical patching environment) enable users to program ML pipelines in the data-flow paradigm.

Fiebrink et al. (2011) used the Weka API in the development of Wekinator, a general-purpose standalone application for applying supervised machine learning. The Weka API is an object-oriented API, written in Java which provides standard implementations of learning algorithms. Wekinator provides a high-level interface to a workflow which that enables users to rapidly create and edit datasets, and to employ these algorithms (and others such as SVM, Dynamic Time Warping) to train and run ML models in real time. It also supports the configuration and mapping of sensor data to end-user musical software, using high-level application pipelines connected through the OSC communication protocol. This end-user programming (Lieberman et al., 2006) approach to IML has been employed in the exploration of user interactions with machine learning in the context of music composition and performance.

The Gesture Recognition Toolkit (GRT) (Gillian and Paradiso, 2014) is an OSS, cross-platform C++ library aimed to make real-time machine learning and gesture recognition more accessible for non-specialists. GRT adopted core design principles which include:

- Simplicity and accessibility, provided by a minimal code footprint and consistent coding conventions.
- Flexibility and customizability, supported by modular architecture structured around the metaphor of a real-time multimodal data pipeline.
- A supporting infrastructure offering a wide range of algorithms and functions for pre- and post-processing, feature extraction and data set management.

Although GRT provided benefits and advantages over more typical ML development environments (e.g., Matlab) it remained difficult to utilize by people who had not the C++ and software engineering skills for the lower-level parts of the code. Nevertheless, it paved the way for other approaches to ease user adoption. For instance, *ml.lib* (Bullock and Momeni, 2015) is an OSS machine learning toolkit designed for two domain-specific data flow programming environments, Max and Pure Data^8^. *ml.lib* was implemented as a set of modules that wrap up GRT library components (Gillian and Paradiso, 2014) and execute within these environments as external components. Besides GRT's core principles which *ml.lib* builds upon (Bullock and Momeni, 2015), other aspects of its design rationale include:

- enabling users without ML background to experiment with and integrate a wide range of ML techniques into their projects.
- taking advantage of the affordances of data-flow programming environments, including (a) rapid prototyping and (b) multimedia integration, (c) high-level abstraction which hides away threading and memory management, and (d) integrated documentation with interactive examples.
- maximizing learnability and discoverability through “a simple, logical and consistent, scalable interface.”
- providing portability and maintainability through the use of a cross-platform and multi-target technology stack that supports different desktop operating systems and embedded hardware architectures and processors.

Another ML toolkit which builds upon GRT and takes another approach to bridge the gap for ML-non-expert developers is ESP (Mellis et al., 2017). The ESP approach intensifies the domain-specific and adoption orientation through the provision of augmented code examples of end-to-end ML pipelines (e.g., audio beat detection). These examples are written by experts using the GRT library and the OpenFrameworks creative coding framework. This approach makes a few assumptions such as the existence of a community of vested experts willing to contribute their ML design expertise to the creation of augmented code examples. Another assumption concerns the tight coupling of the augmented code examples with high-level GUIs, which is deemed fundamental to the learning of the machine learning workflows.

Other ML toolkits have been designed with usability as a primary concern. For instance, Keras is an open-source deep learning API which, according to the author F. Chollet^9^, was designed for usability and with usability principles in mind—consistent and simple APIs, end-to-end pipelines with minimal number of user actions required for common use cases, and clear and actionable feedback upon user error. The usability-focused innovation in ML API design of Keras led to its recent adoption as one of the main interfaces of Tensorflow ecosystem (Abadi et al., 2016). The Layers API from Tensorflow.js (Smilkov et al., 2019) is modeled after Keras, also building upon the advantages of Javascript (JS)—e.g., WebGL-accelerated end-to-end ML pipelines supporting both training and inference in the browser; predefined layers with reasonable defaults; ease of distribution and deployment; portability, server-side and client-side execution in the browser; the wide adoption and relatively low-entry barrier of the JS programming language for novice programmers.

As part of the Tensorflow ecosystem, Magenta.js (Roberts et al., 2018) is an API for pre-trained music generation models. This library was also positioned to bridge the ML-non-expert developers gap through the provision of an even higher abstraction level. One important design assumption is its abstraction-level; hiding away “unnecessary complexities from developers […] would remove the need for machine learning expertise” (p. 1). Magenta.js employs a transfer learning approach (Jialin and Yang, 2010)—i.e., enables the integration of pre-trained models to trivialize end-user adoption—with model weights, parameters, and description made accessible from an URL (remote js-checkpoints). Magenta provides music-specific data structures such as NoteSequences—an abstract time representation of a series of notes, characterized by attributes pitch, instrument and strike velocity (akin to MIDI). It also provides API objects which wrap up deep learning models for musical application—e.g., music variational auto encoder MusicVAE, MusicRNN, Music Transformer, etc. These are at the core of a growing list of examples—both developed in-house and by the community—of the application of the library to demonstrate cutting-edge deep learning techniques for music generation through a carefully crafted set of interactive example applications with publicly available code.

*ml5.js* is another example of an ML API which also builds on Tensorflow.js. However, *ml5.js* provides an even higher-level of abstraction to empower artists and creative coders. According to Daniel Shiffman^10^, the two main barriers to the adoption of ML which ml5.js aims overcome, are “having to install and configure a development environment and secondly, having to implement low-level mathematical operations and technical code.” *ml5.js* aims to support an accelerated learning experience by providing an integrated set of online resources—e.g., the API documentation linking collections of code examples with relevant applications in the P5.js online code editor, video tutorials. There are introduction to complementary technologies (e.g., javascript, the library P5.js, webservers) and to more challenging programming concepts such as asynchronous operations and callback functions. The code examples which are enable training in the browser employ the Tensorflow Visor, an interactive visualization utility that provides feedback on the neural network training and loss function minimization.

There are common traits to these ML APIs and toolkits which have been created using a domain-specific approach to music technology. They share usability principles and accessibility concerns reflected in the ease of deployment, higher-level of abstraction, constraints to the ML pipeline building. They also share code examples grounded on musical or sound applications, which also show interactivity playing a fundamental role in improving the accessibility and understandability of the ML API for ML -non-expert developers. For instance, in the case of *ml.lib*, this happens through the affordances of the interactive data-flow environments, which are effective at conveying information structures with a pipeline metaphor (Green and Petre, 1996). In ESP, the GUIs are not just API client code; rather, they play an essential role in the illustration of the main functional code blocks and in enabling the training and inference workflows supported by an augmented code example ML pipeline. In ml5.js, ML model pipelines are built according pre-defined configurations based on the specific task (e.g., classification or regression). In Magenta, code examples feature pre-trained models such as recurrent neural networks for melody generation and musical accompaniment, automatic music generation from performance MIDI datasets, and for interpolation between melodic lines, drum sequences, and music styles.

ML APIs can influence the features and interaction style of the resulting applications. They can also impact the developers' working processes and experience with ML. However, the challenges and experience of developers working with ML APIs remain under-explored, particularly for the development of IML systems for creative and musical technology.

## 2. The RAPID-MIX API

**Figure 1:**
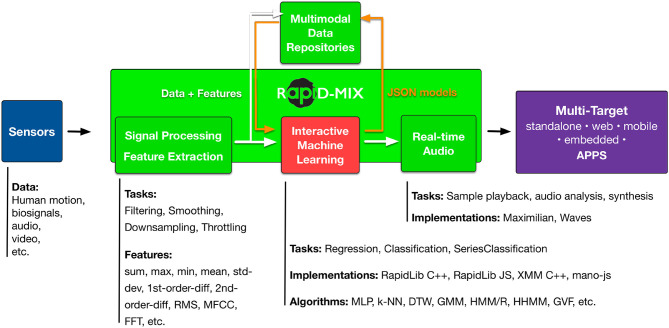
General structure of the RAPID-MIX API.

The RAPID-MIX API is a toolkit comprising ML libraries and learning resources (Figure 1) that were designed and implemented in the context of RAPID-MIX^11^, an EU innovation project focused on the creative industries. The RAPID-MIX project stakeholders identified a variety of potential scenarios for an API that would support rapid prototyping with interactive machine learning for creative and music applications. The API was intended to support both product development by small and medium companies (SMEs)—including music technology companies, e.g., ROLI^12^, AudioGaming^13^, and Reactable Systems^14^—as well as by individual developers working in creative and musical technology. Domains of use included education, games development, music technology, e-health, and sports. Use cases included potential creative products from the previous domains where sensor-based interaction, expressive multimodal control, mapping and rich audiovisual output could benefit from a flexible rapid-prototyping API. Target environments could be desktop, mobile or web apps, or embedded processors.

### 2.1. User-Centered Infrastructural Software

The design of the RAPID-MIX API followed a user-centric approach, where different stakeholders were engaged early and throughout the process, including academics, end-user developers—people developing systems for personal use (Lieberman et al., 2006)—and professional developers working in creative and music technology companies. The design of the API targeted students, “hackers,” and “makers” who might wish to develop other new technologies using ML. The RAPID-MIX API aimed to explicitly support IML approaches to systems development, in which developers can iteratively create, curate, and modify supervised ML training sets in order to influence model behavior.

Design iterations were informed by lightweight formative evaluation actions (Bernardo et al., 2018) using techniques such as direct observation, interviews and group discussions in workshops and hackathons, and remote Q&A sessions between API designers and users. This work contributed to a better understanding of the needs, goals and values of the target users of the RAPID-MIX API, which spanned a breadth of software development skills, experience, motivation, and technical approach expected from creative and music technology developers. Most notably, target RAPID-MIX API users had little to no prior ML expertise, which strongly informed the design considerations and trade-offs.

Early uses of the RAPID-MIX API by creative developers included the integration of the IML workflow in ultra-low-latency audio applications in embedded systems, driving visual parameters of video jockey apps with audio and multimodal feature analysis, and browser-based audio synthesizers and sequencers using real-time sensor data (e.g., Leap Motion^15^ hand pose data, Myo^16^ electromyography and inertial measurement data, BITalino^17^ data), applications for custom control of 3D mesh animation, the rock-paper-scissors game, etc. Additional illustrative use cases have been developed by participants of this study (section 4.1) which include the creation of commercial musical software products.

### 2.2. API Design Decisions and Architecture

The RAPID-MIX API aims to facilitate rapid prototyping by developers in ways that are similar to how Wekinator (Fiebrink et al., 2011)—a popular GUI-based tool for creative IML—supports its users. For instance, it aims to minimize the number of actions a user needs to take to develop a working prototype (see Listing 1). Users need only to create an instance of an ML class, train a model, and run it on new data; no additional setup is required. Further, default values that support common use cases are provided for all configurable algorithm parameters so that developers do not need to make initial choices when building a working system. For example, by default the multilayer perceptron (MLP) has one hidden layer and the same number of hidden nodes as input nodes. If users find that this architecture is not suited to their needs, they can use additional functions to adjust either of these parameters.

**Listing 1:**
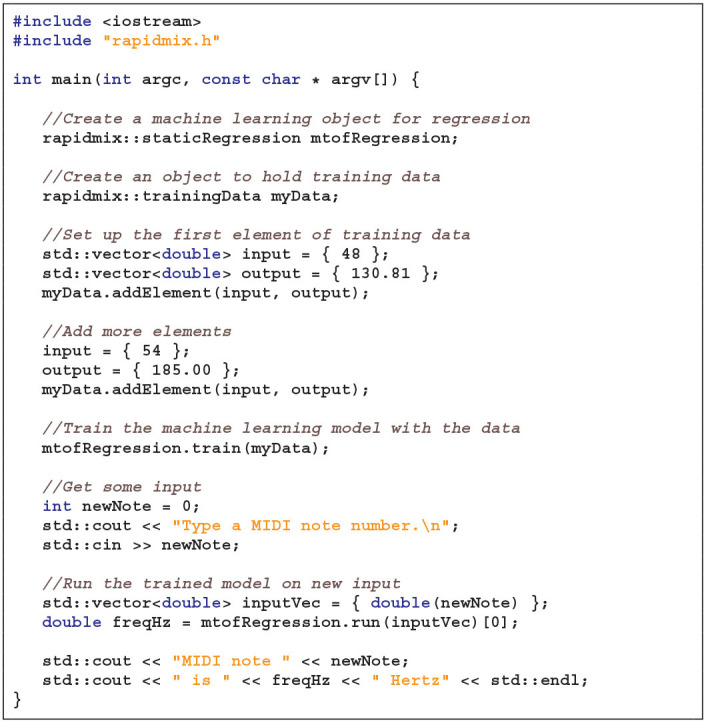
RAPID-MIX API “Hello World” example in C++.

The RAPID-MIX API aims to focus developers' attention on their intended system design, rather than on ML algorithms or architectures. It presumes an ML architecture common to many applications involving real-time audio, visuals, or sensor-based interaction, in which inputs (i.e., vectors of values representing current sensor or media features) are sent to a trained model or set of models, which in turn produce a vector of outputs that are passed to some other real-time process. For instance, the sensor values generated by a specific hand position sensed with a Leap Motion (inputs) might be associated with a set of parameters for an audio synthesizer (outputs). The designer of a new system should primarily be focused on reasoning about what inputs are expected, what outputs are desired, and whether the current trained model is sufficient given these criteria—not about which specific ML algorithm should be used.

The API therefore makes a distinction between two types of design tasks (classification or regression tasks, corresponding to the assignment of discrete categories or continuous numerical values), and, separately, between two types of inputs (static or temporal data, which for instance would correspond to a hand position or a hand movement over time). The core API classes reflect this structure, for example “rapidmix::staticClassification.” When we must ask users to learn ML terminology, we take care to use standard terms, such as classification or regression.

The RAPID-MIX API wraps new and existing supervised ML algorithms in a modular fashion, allowing them to be configured for different use cases. Specifically, it includes FastDTW (Salvador and Chan, 2007), XMM (Françoise et al., 2013), Gesture Variation Follower (Caramiaux et al., 2014), k-nearest neighbor, and neural networks. These algorithms were chosen to have low training and run times, and the ability to create expressive models from small training data sets (Fiebrink et al., 2011). They have been integrated as module components and made available in the different API subsets (i.e., RapidLib C++, RapidLib JS, XMM C++, mano-js).

Further classes are provided alongside the ML classes. For instance, there is a single class that provides an API for creating and managing sets of training data. This class is compatible with all of the ML classes, allowing users to switch easily between ML algorithms while keeping the same training set. The API provides signal processing and feature extraction functionality for audio and multimodal sensor data, ranging from basic processes (e.g., low-pass filters or RMS values) to audio segmentation and Mel frequency cepstral coefficients (Logan, 2000). It also provides methods for serializing and deserializing training data and trained models using JavaScript Object Notation (JSON).

The RAPID-MIX API is designed to allow users to test, train, and use algorithms on multiple devices and move easily from one device to another. In order to support native desktop, browser-based, mobile, and embedded applications, the API is available in both JavaScript (JS) and C++. The JS API provides client-side and server-side libraries, targeting desktop and mobile browsers. C++ is intended for low-level audio and media developers, native mobile apps, and embedded processors. It has been tested in openFrameworks and JUCE, as well as on Raspberry Pi and Bela embedded hardware (Bernardo et al., 2018).

The need to support such a wide range of users and use cases inevitably led to compromises. One substantial subset of the RAPID-MIX API functionality, RapidLib, includes the functionality for classification using k-nearest neighbor, regression using multi-layer perceptrons, temporal classification using dynamic time warping, and signal stream processing. The RapidLib subset is implemented in C++ and transpiled into asm.js using Emscripten (Zakai, 2011). This approach provides advantages such as reduced development time, a great deal of consistency across C++ and JS versions of API components, and more efficient JS code (Zbyszyński et al., 2017). The compromises that such approach entails is that RapidLib generated asm.js code base is opaque to JS users. Furthermore, some features that are idiomatic to one specific language such as multithreading and JSON support are difficult to implement across two languages.

**Figure 2:**
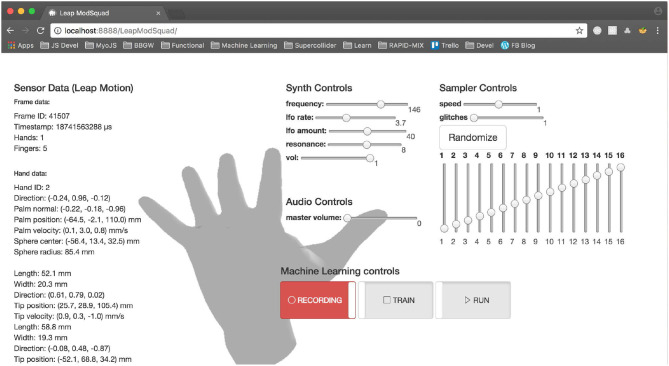
RAPID-MIX API demonstrator for a regression model mapping Leap Motion sensor data streams to a multi-parametric browser-based sampler and synthesizer.

In addition to the software libraries, the RAPID-MIX API comes with documentation and examples to help users learn about and experiment with the API. Working examples show users exactly how to implement common use cases, such as using a Leap Motion sensor to control multiple synthesis parameters (Figure 2), or applying classification to an incoming video stream. In addition to describing the API, the documentation also explains many relevant concepts behind the API, such as the application of filters to multimodal input, what a machine learning model is, or how to construct a training data get. Interactive online examples are provided so users can experimentally apply IML workflows to data created in real time in the browser.

In contrast to other ML APIs, the RAPID-MIX API does not provide built-in functionality for quantitative analysis of the performance of trained models. The RAPID-MIX IML workflow is intended to develop quick prototypes and allow users to subjectively evaluate whether the resultant model is performing adequately, by applying the trained model to new data in real-time and observing the results. When training data are provided interactively, as in the main workflow encouraged by RAPIDMIX API, such direct observation of a model's behavior on new data is often the most effective way to assess a model's performance (and evaluation using more conventional metrics such as cross-validation can be misleading) (Fiebrink et al., 2011).

Listing 1 presents a “Hello World” example of the RAPID-MIX API in C++. Where practical, the same functions are part of the JavaScript API, although obvious differences (e.g., std::vectors) are not duplicated.

## 3. Method

The overall objective of this work was to obtain a deeper understanding about how the design decisions and trade-offs of an API for rapid prototyping of creative technology with IML affect its usability and developer experience. We refined this objective into the following key research questions:

1. What usability issues can we find with the RAPID-MIX API?
2. How do users perceive the RAPID-MIX API design trade-offs and how do these relate to usability and developer experience?

To answer these questions we designed a study with participants who used the RAPID-MIX API in their work and who were asked to report on their experience using an adapted version of the CDs framework questionnaire by Clarke (2010). The questionnaire answers were analyzed using a qualitative approach that is detailed in sections 3.2 and 4.

### 3.1. Participants

We selected participants who had used at least one subset of the RAPID-MIX API within a creative software project. Participants signed a consent form to participate on the study. Our sample set of 12 participants (1 female, 11 males) comprises 6 professional developers working in 3 small and medium-sized enterprises (SME) in creative technology, and 6 creative non-professional developers creating systems for their own personal use (students of different levels, spanning undergraduate, masters level and PhD students; see Table 1). Participants had varying software development experience, varying ML experience (from none at all to experience with frameworks such as tensorflow, scikit-learn, etc.). Participants had used different subsets of the API (i.e., RapidLib C++, RapidLib JS, XMM C++, or mano-js) for varying amounts of time (for less then 1 month to a little more than a year). Some participants used the API in personal projects or proofs-of-concept outside the commercial sphere; other projects were developed for commercial purposes in a professional context.

**TABLE 1:** Listing of study participants.

**ID**	**Software dev. experience (years)**	**ML experience**	**API subset used**	**Time using API (months)**	**Use (personal, commercial)**
P01	4	Some	RapidLib C++	8	Personal
P02	1	Some	RapidLib C++	11	Personal
P03	6	Some	RapidLib JS	1	Commercial
P04	6	Some	RapidLib JS	6	Commercial
P05	14	Some	XMM C++	>12	Commercial
P06	5	Some	RapidLib JS	5	Personal
P07	3	Some	RapidLib JS	<1	Personal
P08	5	None	XMM C++	1	Commercial
P09	5	None	mano-js	<1	Commercial
P10	1	Some	RapidLib JS	<1	Personal
P11	1	Some	RapidLib C++	>12	Personal
P12	7	None	XMM C++	6	Commercial

Commercial products created by participants include: a JS front-end component that integrated the IML workflow into a commercial biosignal analysis toolkit for rehabilitation engineers working with patients (P03, P04); an intelligent drum sequencer for iOS with custom gesture activation (P05, P08, P12); and a software-as-a-service that coordinates sonified movement workshops and soundwalks, using the multimodal and multimedia capacities of these collective events attendees' mobile devices (P09).

### 3.2. The Cognitive Dimensions Questionnaire

We employed an adapted version of the CDs framework questionnaire (Clarke, 2010) as our research instrument, which appears in Appendix A. This questionnaire has been developed to support a comprehensive and systematic understanding of participants' experiences with the API, broken across several different dimensions. Clarke's questionnaire provides several benefits over the original CDs questionnaire, as it is tailored for API evaluation, and it also has an increased focus on learnability (Clarke, 2010)—i.e., introducing additional dimensions including Learning Style, Penetrability. We were inspired by prior studies that fine-tuned Clarke's questionnaire to specific domains—e.g., Watson (2014) introduced high-level groupings of dimensions for a more effective distillation of results for improving API documentation planning; Wijayarathna et al. (2017) aimed to evaluate aspects of their API that were specific to cryptography by introducing additional dimensions (e.g., End-user protection, Testability). We have also adopted some of these dimensions with minor changes. Table 2 summarizes the 14 dimensions used in our questionnaire, grouped into four high-level themes. Each dimension was addressed by several questions (Appendix A).

**TABLE 2:** The adapted Cognitive Dimensions framework used in our study.

**Learning**	
Abstraction Level	Magnitude of abstraction and style of abstractions of the API
Learning Style	Learning requirements and style encouraged by the API
Penetrability	Ease of access, retrieval, exploration, analysis, and understanding of the API components
**Understanding**	
Consistency	Similar semantics are expressed in similar syntactic form
Role-expressiveness	Purpose of an API component is readily inferred
Domain Correspondence	Clarity of domain mapping of API components
**Usage**	
Working Framework	Size of conceptual chunk or amount of context necessary to work effectively
Elaboration	Extent to which API must be adapted to meet developer needs
Viscosity	Resistance to change in refactoring
Premature Commitment	Constraints in the order of implementing API code
Error-proneness	Error incidence, recoverability and support
**Application**	
Work Step Unit	Amount of programming task completion achieved in a single step
Progressive Evaluation	Work-to-date can be checked at any time
Testability	Types of evaluation and assessment metrics that are adopted

We first delivered a pilot version of our questionnaire to two participants. This version was longer and closer to the original version by Clarke (2010), and we received complaints about its length. We therefore shortened the questionnaire by removing some redundancies. The final questionnaire was delivered online, on paper, or through in-person or remote interviews, due to the geographical spread of the participants.

## 4. Results

In this section, we report our findings about each of the dimensions included in our questionnaire. We employed content analysis using NVivo to analyses responses. We adopted a deductive analytical approach in which we used codes based on the CDs framework and on the higher-level themes of Table 2, and on an auto-encoding analysis performed with NVivo.

We also tried to find correlations between the variables Software Development Experience, ML Experience, API subset, and time using the API, in the closed-end questions of each dimension (e.g., Q1—perceived level of abstraction, Q8—learning experience, Q11—experience with amount of context, etc.; Appendix A). Given the size of our sample, we ran Pearson's chi-squared test with Yates correction, and Fisher's exact test. We found no support for contingency between those variables in the dimensions' quantitative results as none of the tests yielded statistical significance.

### 4.1. Abstraction Level (Q1–Q2)

Questions pertaining to this dimension aimed to investigate the appropriateness of the abstraction level of the RAPID-MIX API. We asked how appropriate the abstraction level was for participants' development needs and why (Q1, Appendix A), and whether participants felt they needed to know about the API's implementation details (Q2).

In responses to Q1, 7 of 12 participants found the overall API abstraction level “just right,” and 5 of 12 found it “too high level.” No one found it “too low level.” Five of the 7 participants who had used the API for longer than 2 months found the abstraction level “just right.” Participants who used different subsets of the API differed in their responses; all the participants using RapidLib C++ (4 participants) or mano-js (1 participant) considered these had the right abstraction level, and 3 of 4 participants using RapidLib JS considered it too high-level.

Participants who found the abstraction level just right described how the abstraction level enabled them to achieve their development goals (P01, P02, P06, P11). These included rapid prototyping (…“I was able to do rapid prototyping, rapidly!”, P06), simple implementations (“for a quick and simple implementation the abstraction level works well”, P03), and proofs-of-concept (P04). Participants also referred to the positive development experience the API provided, having found it “extremely easy to use in C++, which is usually a very confusing language” (P02), or non-obtrusive to the creative process— “I was able to implement most of the RapidLib functionality without losing my creative flow” (P06). P04 indicated that the API “facilitates the final programmer use” and saved her a lot of time by preventing her from having to handle implementation details.

Participants who found the RAPID-MIX API too high level (P03, P05, P07, P08, P10) experienced problems mainly because they needed further understanding of lower level details—“when I tried to learn a little more, knowing, for example, which model was being used, I saw the abstraction level as a hindrance” (P03)—or finer-grained control over certain API features—I found that while the algorithms work, I would have liked a bit more control over certain algorithms” (P10). Some participants complained about the lack of transparency of RapidLib JS's high-level objects (Classification and Regression) which prevented them from knowing which algorithms were in use. Because RapidLib JS is transpiled from C++ to asm.js, the source code and algorithm implementation is more opaque than the C++ version.

Participants who stated that they needed to know the underlying implementation (Q2) presented different reasons for this. Three participants (P05, P07, P10) found undocumented algorithm parameters—e.g., k in the k-nearest neighbor algorithm, the number of hidden units in the hidden layers of the multi-layer perceptron—and needed to understand how to use them, so had to look further into the implementation. Some of these participants worked on a product and related their reasons to needing a deeper understanding for customer-facing projects (P03, P08). For instance: “I needed to know what was behind the object. If I am going to make a product or give a chance to a customer to use one of our solutions based on the result of the api, and for some reason, something wrong happens it would be necessary to have a deeper knowledge of the whole object.” (P03).

One professional developer, P05, considered the understanding of the underlying implementation vital—“The API is very concise and there's not much to learn, however choosing the correct parameters is a ‘dark art”' (P05). One participant found the library opinionated (“it is intended to work in a specific manner”) and had to look to the implementation to adapt it to their needs.

Participants who mentioned not needing to know the underlying implementation either mentioned that they already knew it, or, that they had the sufficient knowledge to be able to use the API successfully—“I felt that I needed general knowledge of how regression and classification algorithms worked. However, for my purposes this was enough. I could then just use the API without needing to know the exact implementation.” (P11).

### 4.2. Learning Style (Q3–Q9)

The questions about learning style aim to determine what knowledge was essential to use the API successfully, how much new knowledge participants had to acquire, and how participants went about using API documentation to attain this knowledge.

Participants perceived knowledge of the following ML concepts to be important in facilitating use of the API (Q3): the probabilistic nature of ML (P05); the greater importance of the choice of data in comparison to the choice of algorithm (P01, P05); basic ML concepts such as the difference between regression and classification (P02, P04, P11); the stages of the supervised learning workflow, such as collection and preprocessing of data, training and running the models (P01, P03, P04); and understanding the ML algorithms' implementation and inner workings (P01, P03, P07). They also identified the following knowledge of non-ML topics as useful (Q4): threading and non-blocking async architectures (P06), client/server architectures (P09), deeper programming language knowledge (e.g., using generics) (P12), statistics for modeling data (P05), and practical knowledge about sensors, and human-computer interaction (P11).

Participants' responses about their learning strategies (Q6) indicated that both novice and experienced developers tended to adopt an opportunistic approach (Clarke, 2007) to learning about the API: they frequently learned by copying sample code and employing hands-on exploration. The more experienced developers appear to have complemented this with a more top-down approach to learning about the API components or architecture.

The majority of participants (9 of 12) indicated that they had to learn “just [the] right” amount to use the API (Q8). These participants defended this response with answers that mentioned the simplicity, ease of use, and beginner-friendliness of the API. For instance, participants wrote that the “code of the API is simple and concise” (P05), that it was “straightforward to use without having to read too much documentation” (P07), and that “I didn't have to learn anything new to use the API and I didn't want to learn a lot to train such simple models” (P02). The other 3 participants stated that the RAPID-MIX API documentation did not provide enough resources to support their learning, particularly regarding choosing appropriate algorithms and their parameterizations for a given problem. P12 wrote “one is left guessing numbers and trial and error exploration, if there's no previous ML experience,” and P01 wanted “more complex examples so that people can try different ML structures.”

### 4.3. Working Framework (Q10, Q11)

Q10 aims to elicit an understanding of the amount and type of information (i.e., “context”) a user needs to maintain or keep track of while working with the API. Eleven participants responded, choosing multiple elements from the list of contextual information (one participant did not respond). The top 5 classes of context identified as necessary by respondents were: API methods (10 of 11 participants), API classes (8), data structures for training and model input/output (7), database (e.g., JSON, XML, CSV, database management service) (7), and types of ML algorithm (6). Less common responses included: local scope variables (4 of 11), system configuration settings (4), app configuration settings (3), global scope variables (2), registered events (1).

When we asked participants to describe this “amount of context” was “too simple,” “too complicated,” or “just right” (Q11), 8 of 12 participants reported it was “just right”. Participant explanations suggest this was driven by the simplicity of the API interface—“not very demanding, inasmuch as methods presented simple syntax. When developing, I didn't usually keep track of them. When problems arose, it was always easy to navigate to tutorial examples and spot where scope or method syntax was not correct.” (P03). Contrastingly, the participant with the most ML expertise conveyed his reasons for what is important about context using more of a conventional rationale around context—“I needed to keep all the context in mind that is relevant to using the algorithm. I guess I don't care that much about the particular data structure that the API expects, so it would be nice to not have to think about that. I don't see how you could avoid that though” (P06).

Two respondents found that the amount of context they needed to keep in mind was too complicated. One of them, the less-experienced, found difficulties with developing architectural support for an increasing number of model outputs—“adjusting the output parameters of my application took a bit of time and thought to figure out what parameters needed to be global and what parameters needed to be local.” (P10). The other respondent, a more seasoned developer, implemented a non-blocking asynchronous threading architecture for making a robust use of the API—e.g., “Training with large amounts of data can take a long time and should be non-blocking e.g., a future. However, it also needs to be cancellable.” (P04)—which entailed the use of a more comprehensive and complex “context.”

Interestingly, one participant referred specifically to training data and its specificities for the IML workflow as part of the ‘context' to be kept in mind—“Often in the Application I would visualize the training data or rely on remembering it. So just being able to save the model without the training data was not useful and caused complexity issues. Especially when the training time of the models is very short and the datasets are small” (P02).

### 4.4. Work Step Unit (Q12)

Q12 asked participants whether the overall amount of code they had to write to integrate the API into their projects was too much, too little, or just right. Eight of 11 participants mentioned that their experience was just right.

The remaining participants answered that they had to write too much code. Their explanations identified several tasks that appeared to require too much code: (a) validation, (b) data management, (c) concurrency management, and d) management of model outputs. Three participants mentioned validation code as necessary to make their application safe and robust—e.g., “there's not too much error handling, or check on the data format/range” (P12). Two participants referred to concurrency—e.g., “not ‘too much' code directly related to the API, just too much boilerplate wrapper code in order to use the API successfully in the context of a large multithreaded mobile app with GUI and audio” (P05). Three participants mentioned having used an extensive amount of code for creating data structures and performing data management in the application. For instance, “I had to write lots of code for formatting training data, I feel like the API could have given an interface for recording, building and editing data sets rather than needing to be given the whole dataset at once or relying on user-written C++ vector functions to edit training data” (P02).

### 4.5. Progressive Evaluation (Q13)

Q13 asked participants about the amount of work needed to evaluate progress in using the API. Notably, though participants knew the questionnaire was focused on evaluating the API itself, we found that the majority of responses related to the task of evaluating progress of the IML workflow outcomes (i.e., the quality of training data, the training process, and the model results) rather then just progress in establishing a functional pipeline.

Participants identified the simple API interface as facilitating progress evaluation—e.g., “It was very easy to evaluate the integration of the API with my application. Because of the simple user interface of the API, I knew exactly what to expect from each method of the API.” (P02); “there is very little code required to use the API. Evaluating the performance of the tool, selecting the source data inputs, choosing a frame rate, ensuring orthogonality took up 10% of our time.” (P05).

Responses that expressed difficulty in evaluating progress shared some common themes. For instance, respondents complained about the lack of integrated visualization tools—“I evaluated my progress in using the API by implementing visualizations in D3 […] I would probably like to minimize the amount of time spent on visualization code” (P07). Others complained about the lack of functionality to provide feedback about model accuracy improvements—“There's no proper visualization of improvement, one is left with the trial and error to determine if the classification is improving or not, and no information on how good/bad it is.” (P12). One participant referred to the high abstraction level as a hindrance for progressive evaluation—“Because some of the functionality of the API is hidden for advanced or personalized use cases, I wasn't completely sure about my own progress” (P06).

### 4.6. Premature Commitment (Q14–Q17)

Q14–Q17 examined how participants perceived the level of premature commitment required by the API—i.e., the need to make certain decisions too far in advance, and inflexibility in the order in which decisions had to be made.

Eight of 12 participants reported that they were forced to think ahead and make early decisions (Q14). Most participants found it necessary to make early decisions about data sources and preprocessing, data structures for inputs and outputs and their dimensionality, and the state machine architecture that supports switching between the modes of training and running the models (Q15). Some of the more advanced users, or users with more complex requirements for commercial product implementations, referred to planning the integration of the API components according to specific aspects of their use case—for instance, within a client-server or concurrent architecture.

### 4.7. Penetrability (Q18–Q23)

Questions about penetrability aim at understanding the degree of ease with which developers could access information about API components, and explore, analyse and understand their working details in order to achieve specific development goals.

Eight participants encountered some difficulties in finding necessary information about API details (Q18, Q19), indicating that the documentation of API subsets was insufficient. Most of these respondents had, at some point, finer-grained implementation requirements for which necessary details about the API became hard to find. Seven participants indicated having to learn about specific ML algorithms and parameter configurations (Q20). Some participants learned about these as they worked—e.g., “Online tutorial materials and examples were very helpful. However, should deeper potential of the API be explored, I can't say that all questions would be easily answered.” (P02); “As my own knowledge [of IML] progressed I would have liked to be able to find out more detailed information about the neural network and how it performed the regression analysis” (P03).

Participants reported working to improve their understanding of the API (Q22) mainly through the process of trial-and-error exploration (5 participants) and by reading through the API source code (4 participants)—“Largely through trial and error I began to get a sense of how the regression model worked” (P08); “By breaking it, using intellisense to find functions that were templated to exist, but did not have implementations in some models, so I started reading more of the API's source” (P01). Some participants reported needing to use direct communication with the API developers (P01, P06, P09) and resorting to external documentation resources (P05).

Four participants believed the API and its documentation provided enough information for their needs (Q23), found easy access to that information (Q19), and that there was no lack of information about details (Q18). Most of these participants either had simple goals and remained at a high implementation level, or their exploration was technical-driven rather than design-driven—“My original interest [lay] in the C++ API, but resources and adaptation to the final product needs made me shift toward Javascript, which had magnific learning materials” (P04). Some participants admitted not understanding the working details but were satisfied working with a “black box”—e.g., “I didn't fully understand then. The results were adequate enough for our application” (P09); “I had no knowledge of the implementation details or how the API is generally structured apart from what was obvious from the code examples” (P05).

### 4.8. Elaboration (Q24)

Q24 asked about the ways that participants had adapted the API to fulfill their design goals (if any). Five of 12 respondents used the API “as-is.” Five others reported having wrapped the API in adapter classes to add the necessary functionality to overcome specific limitations of the API. Three of these respondents had added error handling and support for asynchronicity.

Two participants reported having forked the API and changing the library file structure. One respondent hacked the API to improve the learning capacity of the default regression object. His hack approximated the functionality provided by an undocumented MLP parameter—“The hack was to increase the dimensionality of the input vectors by duplicating their content. This would artificially increase the number of hidden units and allow the model to learn more complex patterns” (P06). No respondents reported trying to derive classes or override class methods.

### 4.9. Viscosity (Q25)

Q25 aims at understanding how is easy it is to make changes to code that uses API calls. Seven of 12 respondents mentioned it was easy and two mentioned it was very easy to make changes to API integration code (Q25)—“Easy, there was barely any code to write to implement the API.” (P02); “Very easy. The interface is minimal and the actual parameters that one can change are few” (P12). Three respondents mentioned they did not need to refactor their code. The other two respondents described challenges around understanding the code in the context of refactoring it—“Easy as I wrote the code […] When debugging issues though, I needed to check examples a lot to understand the described Test-Train-Run structure that I needed to implement. As in ‘to train only once and not to run the model when testing or training'.” (P01); “It was easy but needed a lot of understanding of the code.” (P08). One participant referred to the growing amount of outputs as a difficulty for change—“As the amount of output parameters grew I found it sometimes difficult to keep track. Otherwise it was very easy” (P11).

### 4.10. Consistency (Q26)

Q26 asked participants if they noticed API elements that offered similar functionality, and whether the differences between them were clear (Q26). Five of 11 respondents mentioned having noticed consistent method names across classes. Three of the aforementioned 5 found lack of clarity between certain API classes—e.g., “Model set, Regression and Classification. The difference between these objects was not clear. The implementation[s] were all very similar and it was not clear which one to use” (P02). There were also issues around the use of the different kinds training data structures. The other two who noticed consistency of methods felt they understood the differences between them. For instance: “I like that there were a train, run functionalities in the code as this help me understand the models in similar way apart from the inner workings of course” (P01). The remaining respondents (6 of 11) did not noticed such similarities; one participant did not respond.

### 4.11. Role-Expressiveness (Q27–Q29)

We asked participants if it was easy to read and understand code that uses the API (Q27), and whether it was easy to know which classes and methods to use (Q29). We obtained unanimous responses to both questions—“Everything was very easy to interpret.” (P02); “Code is pretty self-explanatory and comments are concise enough” (P04) “Classes methods are efficiently named to understand what they are doing” (P08).

### 4.12. Domain Correspondence (Q30–Q32)

Questions about domain correspondence aim to determine whether API classes and methods map easily onto the conceptual objects in the users' implementation.

We obtained unanimous positive responses about the ease of mapping the API code into developers' conceptual objects (Q30). Two respondents provided reasons that related the simplicity of the API interface and the IML workflow to the ease of mapping to domain and conceptual objects of their implementation (Q31)—“the simple user interface made prototyping very quick making building a conceptual idea very easy and simple.” (P02); “I think because the training and the recognition phase is the same workflow, it's easy to come up with concepts that match both.” (P07).

Participants seemed to have had a particular understanding of what was meant by the “mapping” of the API to an application domain (Q30); the majority of responses mention mapping API objects to classification or regression tasks, or to the IML workflow tasks. Most likely, participants have understood ML learning functions such as classification and regression, as enablers of functional mappings between domains (e.g., mapping gesture to sound, visuals, and discrete application events). This seems to be confirmed by the results of asking participants to provide examples of conceptual objects (Q31); only a few participants were able to refer to conceptual objects that did not overlap directly with ML domain concepts—“Once I had a clear idea how I wanted to activate certain functionality, this made the process easier for me.” (P01). “Because the API enable separation between “recording” the data (i.e., training) and estimating the probabilities of category membership for an unknown example (recognition)” (P03).

### 4.13. Error-Proneness (Q33–Q36)

Questions about error-proneness aimed to elicit the participants' experiences with encountering and recovering from errors in their use of the API.

Eight of 10 respondents reported that they had used the API incorrectly (Q33). Errors included: using an inconsistent number of data features between training data sets and test data sets (P05, P06, P09), using malformed data (P04), using labels inconsistently (P12) or malformed JSON (P05, P08), using a large-size training datasets which caused a crash (P11), attempting to predict from a model in an untrained state (P02), and using a higher-abstraction level object as a primitive (P02). Many of these incidents were caused by limitations in input validation of API methods.

Four of these respondents indicated that the API did not provide sufficient help to identify misuse (Q34)—e.g., no error messages, some “undefined behavior” output. Participants reported having experienced crashes of the API-client application without any notification with the subsets XMM C++ (P04, P10, P11) and XMM JS (P09). One participant resorted to logging (P09) and contacted the API developers directly to find and resolve the issue.

Most respondents indicated they were able to find a way to correct their use of the API (35). For instance, where participants encountered errors due to lack of input validation, they adapted the library to implement validation (P05, P12). Other participants simply became more aware of problems and more careful (e.g., in structuring the training data, choosing the correct dimensionality of inputs and outputs, validating model state, etc).

### 4.14. Testability (Q37–Q39)

Questions about testability aim to determine the types of evaluation and assessment metrics that were adopted by participants as they used the API and concluded their implementation of integration code.

Most participants indicated having used subjective evaluation to assess the results of the trained models (9 of 12), with criteria such as correctness (3), cost (3), decision boundary characteristics (1). Several participants referred to other criteria such as expressivity (1) or more creative ways of evaluation—e.g., “No testing was done on the models, just eyeing up the output and judging it creatively whether it works or not for the desired output” (P01). One participant mentioned having used seam tests for assessing training data. One participant did an objective accuracy evaluation of the models built with the API using unit tests with another ML library.

Seven of 11 participants found the API did not provide guidance on how to test the resulting application. The remaining respondents did not look for guidance for testing—e.g., “We tested very informally since there's no effective way to test more objectively” (P12).

## 5. Discussion

According to Clarke (2010), the CDs inspection can tell whether there are significant differences between what an API exposes and what a developer using the API expects. In this section, we use the results of applying the CDs questionnaire with RAPID-MIX API users to discuss design trade-offs with respect to developer experience and ML API usability. We use these insights together with our experience designing the RAPID-MIX API to provide recommendations for the design of ML APIs for prototyping music technology.

### 5.1. ML API Design Trade-Offs in Relation to Learnability and Understandability

Results indicate that the RAPID-MIX API qualifies as an ML API with a high or aggregate *abstraction level*. The high *abstraction level* is supported by its minimal surface area comprising a small number of classes, methods and parameters. These elements have been subsumed into a simple conceptual model of high-level design tasks and basic data structures. An ML API has direct *domain correspondence* if ML is considered its domain of correspondence. In the understanding of most users, RAPID-MIX API entities map directly onto ML learning tasks.

The high *abstraction level* appears to be consistent with the *learning style* of the RAPID-MIX API, which is more of incremental and step-wise. Both novice and experienced developers reported an opportunistic learning approach (e.g., having hands-on exploration and progressing through code examples, exploring, changing or copying sample code to their projects). Arguably, given that ML learning tasks and the algorithms require extensive description from API providers and learning from the users, this indicates that the learning and assimilation of ML concepts was successful. ML APIs with these characteristics can provide ML -non-expert users with adequate scaffolding for a more satisfactory and forgiving learning experience.

However, more experienced developers reported to have complemented their learning strategies with a more systematic, top-down structured learning approach to the components and architecture of the API. More advanced developers and more technically complex scenarios might require the flexibility and control that a lower-level ML API with more primitives, more ML algorithms and more exposed parameters for finer-grained control can provide. We found that a few respondents, the more experienced developers or the ones who had specific implementation requirements (e.g., finer-grained control, strict end-user concerns within customer-facing projects) needed to go “beyond the interface” to inspect the API source code and learn more about underlying ML algorithms. In that exploration, a few of them found useful parameters that had not been exposed. This finding informed a subsequent re-design to expose the parameters.

In scenarios of exploration and intuition building about ML, ML APIs with surface-level *penetrability* may appear to provide everything that is required to enable successful use and integration with client application code. Nevertheless, surface-level ML APIs may allow “black box” approaches in its application and use. We found that the RAPID-MIX API was no exception to this. As developers build up knowledge and understand the IML workflow, which ML tasks to apply, or the number of inputs and outputs to use in a ML pipeline, they may seek to push forward their understanding of a ML model behavior. They may engage in a deeper exploration and experimentation to learn about the ML API intricate working details, such as the impact of choice of underlying ML algorithms and parameter change.

In the RAPID-MIX API, the overall *penetrability* is mostly sensitive to context and to implementation needs. with different RAPID-MIX API subsets providing distinct levels of *penetrability*. There were cases of deeper exploration fraught with fragmented documentation, and unclear dependencies between API primitives and abstractions. This gives the RAPID-MIX API a core *consistency* rather than full *consistency*. These issues affect the perceived *consistency* of an ML API, and consequently, its learnability. For instance, some participants resorted to external resources to understand ML concepts and algorithms, which may be considered resorting to a top-down approach to learning ML.

Different areas of an ML API may have distinct levels of *role expressiveness* which also affects its *consistency*. In most cases, the purpose of the RAPID-MIX integration code was correctly interpreted and matched user's expectations. Nevertheless, there issues which prevented it to fully match users expectations which gives it a lower *role expressiveness* as an ML API. One opaque subset (i.e., RapidLib transpiled from C++ to asm.js) prevented one user from determining the underlying implementation. As mentioned before, other users found undocumented lower-level methods or lacked configuration settings. The transparency at the level of the ML algorithm—or ML explainability—is another layer that may entangle with the overall ML API *role expressiveness*. However, ML explainability is a current and significant research problem that is out of the scope of this paper.

### 5.2. ML API Design Trade-Offs in Relation to Usability and Applicability

An ML API with a high-level, easy-to-acquire conceptual model can cater well to the opportunistic approach and needs of ML non-expert developers. In the case of RAPID-MIX API, a simple conceptual model based on ML tasks and simple data structures with inputs and outputs, makes it suitable for simple implementations and rapid and pragmatic prototyping with IML. It also helps us to uncover and better understand usage and application of ML APIs by ML non-expert users.

ML APIs with a high *API elaboration* should not impede any kind of user from achieving their design goals. They should enable great flexibility to the more proficient end of the user spectrum, such as the implementation of custom behaviors, custom ML pipelines and parameterization. Almost half of participants reported using the RAPID-MIX API “as-is” to meet their design goals. The other half required further API elaboration (e.g., more ML algorithms, more parameters, better error reporting). This tells that for users with simple goals the RAPID-MIX API was sufficient. Alternatively, it can tell that, for more critical users, or, users with more sophisticated implementation goals, the API was not sufficient.

Arguably, the RAPID-MIX API exhibits a medium level of *API elaboration* as advanced users may use its extensible architecture to extend the API default capabilities with custom implementations. The few participants who extended the API default objects did so using adapter classes to extend the default objects and methods with validation, concurrency, and error reporting. However, these users improved upon base limitations of the ML API. For a user, extending an ML API might defeat the whole purpose of using it in first place. Users who do not expect, or do not have the know-how to extend the existing functionality, might find problematic in doing so. They may opt for using a different ML API or framework altogether, or resort to integrate independent ML algorithms.

Developers integrating an ML API in their client application code need to keep track of the information which enables them to work effectively (i.e., the *working framework*). Interestingly, half of the respondents did not mention ML algorithms as part of their *working framework*. This might reflect a trade-off with the *abstraction level* of the API; or alternatively, the adoption of specific ML API design assumptions (i.e., in the case of RAPID-MIX API, data and use cases on the foreground of users' attention and ML algorithms on the background). The lack of preponderance of the ML algorithm may be unsurprising if it reflects minimal ML requirements or a local *working framework* (i.e., ML API objects and methods, local variables) that suffices for simple implementations. However, the *working framework* may not be entirely or directly represented by the ML API or the scope of the ML API integration code—e.g., extrinsic elements such as the ML training data, or in a global or system-level working framework, client application and system configuration settings, external device data specifications, performance requirements, etc.

In a minimal test (e.g., hello world example, unit tests with a ML API) the *work-step unit* might be local and incremental. Despite the minimal surface area of a ML API, developers may have design requirements that scale the quantity of ML API integration code extensively. In these cases, an ML API can have a parallel *work-step unit*, where the steps to implement and achieve the full design goals are distributed throughout different scopes in the integration code. Given the interactive nature of the IML workflow, the ML API integration code will most likely scale up to comprise multiple and independent code blocks. This was the case with a few of the implementations with the RAPID-MIX API, e.g., asynchronous event handlers for collecting data and building an ML data set on the fly, for triggering evaluation of new data, or persistence to data repository. ML API integration code may also require the instantiation of multiple auxiliary objects that interact together (e.g., GUI, data management, validation, concurrency), which make using and understanding more challenging.

Similarly, an ML API may support a *progressive evaluation* of the integration code at local level, functional chunk (that is, after the implementation of certain groups of tasks, such as setting data structures and training data set, or after the train and run methods), or parallel components (i.e., multiple and independent code blocks). The majority of respondents reported needing a fully functional pipeline and to experiment with IML workflow in order to check progress on the overall implementation task with the RAPID-MIX API. An ML API may support *progressive evaluation* at parallel components, as it requires a fully functional implementation and interaction between different ML API objects.

An ML API that presents the user with a small number of choices about how to accomplish design goals with minimal implementation differences between alternatives, can expose a minor and reversible level of *premature commitment*. The RAPID-MIX API also has a low level of *viscosity* that allows users to easily make changes and refactor integration code. This is consistent with the notion that raising the *abstraction level* reduces *viscosity* (Green and Petre, 1996); low *viscosity* is also supported by the API small-surface area. Such ML API qualities invite a trial-and-error exploration and an opportunistic approach, and are supportive for ML-non-expert users.

The RAPID-MIX API situates at a medium level of *error-proneness* given the reports about recurrent issues of misuse, error support and recoverability. These findings indicate opportunities and the direction for technical improvements, such as providing more robust validation of inputs, and better communication of error status through error messages.

Concerning testability, the RAPID-MIX API promotes more of a direct and informal evaluation using subjective criteria. This confirms its alignment with the IML approaches that the API is inspired on. In any case, most developers seem to be unaware of different methods or evaluation alternatives, and seem find the concept difficult to articulate. Also noted was the lacking of guidance about evaluation alternatives, which seems to require specific ways to be successfully transmitted, such as with richer media.

### 5.3. Recommendations for the Design of ML APIs for Prototyping Music Technology

1. *Adopt a user-centered infrastructural software design approach*—find domain-specific core test applications early on which might be compelling to users and help them to understand, and that can inform the ML API core design features (Edwards et al., 2003). In the music technology domain, these could consist of, for instance, a range of new sensor-based interaction applications with complex mappings to music processes (e.g., the main focus of RAPID-MIX API, GRT, and related toolkits), automatic music composition applications (e.g., Magenta), or other types of automation for music production environments. These applications can help to determine many of the ML API design features such as the surface area, its elaboration level (or extensibility) or abstraction level.
2. *Reduce the skills and usage barriers with a minimal code footprint and reasonable defaults*—design the ML API abstraction level to lower the entry barrier for ML-non-expert-users by abstracting details away. Design for improved readability, efficiency and reduce cognitive load with terse ML API code and minimal boilerplate. Users starting with ML API functions should experience a default or typical behavior with default parameters. For instance, RAPID-MIX API offers a high abstraction level, in which ML tasks are high-level objects and data structures are simple arrays. This contrasts with the abstraction level of Tensorflow and GRT, with tensors as data structures or low-level math operations. Reducing the complexity and the number of possibilities of building ML pipelines can accelerate the immediate engagement with the ML algorithm and data, both programmatically and via an IML workflow. This can foster a bottom-up learning style, and provide an increased focus on their design goals.
3. *Facilitate the first contact through an immediate hands-on-code experience*—minimize the cognitive load associated with installation issues to accelerate the first contact at structuring an ML pipeline and fully experiencing an IML workflow for a musical application. Users find it difficult to adopt a tool if they are not able to see it working quickly and providing compelling results, and the installation steps can drastically undermine the developer experience. ML APIs such as tensorflow.js, ml5.js, and the RAPID-MIX API, which offer “zero-install” access to model building and inference in a browser environment can be very compelling for novice users to start with. Similarly, users can benefit from plugins which wrap up ML API components and ease the integration with environments such as Max, Pd, or OpenFrameworks.
4. *Provide adequate conceptual scaffolding for the ML API code*—help the user build an adequate mental model for the ML integration code using different abstractions, if possible from the domain of application, such as end-to-end pipelines, modular building blocks, and training and inference workflows. This can help users to better understand, not only the alternatives which the API makes available (i.e., ML algorithms, objects, and data structures) but how they fit within the working framework required to accomplish their design goals when building musical applications. ML API users building intelligent music systems will develop a working framework of how to set integration hooks between the inputs and outputs of an ML pipeline and areas of the client code (e.g., the controller data streams, the UI event handlers, the audio engine).
5. *Provide many code examples of real-time interactivity between user, data and ML algorithms that can be applied to musical processes*—provide support for the development of intuition and basic understanding with an experiential approach and contrasting ML API code examples that gradually disclose complexity. This will provide users with a smooth learning curve and experience to building ML pipelines and workflows for musical applications. A real-time IML workflow where the end-user creates, curates, and modifies training data iteratively to build ML models mapped to musical parameters, and steer their behavior based on direct observation, trial-and-error and hands-on-exploration, can yield a smaller gulf of execution and evaluation (Norman, 2013) than other workflows. Code examples can support opportunistic approaches—i.e., hacking, appropriation, e.g., ESP (Mellis et al., 2017)—to ML systems development, which might be more enticing to novices or aligned with the goals of rapid prototyping musical applications. Novice users tend to use code examples as the basis and starting point of their music technology creation, so they might be written as building blocks.
6. *Design your API documentation with relevant ML terminology and curated external resources*— design the documentation with an adequate level of penetrability to support an effective learning experience—e.g., navigation, content structure, links to API code (Meng et al., 2019). Limit the use of specific ML terminology conforming with standard terms, while aiming “at the most common case”—e.g., the *Glossary of Terms* (Kohavi and Provost, 1998)—applied to the music domain. For example, understanding the meaning of classification both as an ML task and musical design task (e.g., musical gesture recognition) may lead to cognitive benefits—standardization for improved memorability (Norman, 2013), a general principle which claims usability can be increased by having to learn about a term only once, which potentially lowers the barrier to participation. Documentation includes code commenting practices and the curation of links to third-party content which can provide competent and alternative means of explanation—broader, deeper, or more interactive and engaging (e.g., Youtube, StackOverflow, online playground tutorials).
7. *Error reporting, progressive evaluation and feedback mechanisms*—reduce the evaluation gap of the ML API integration code and help users to recover from errors by providing them with error messages for the relevant parts of the ML API. Most errors identified during usage of RAPID-MIX API were related to ill-structured data sets, and inconsistency in labeling, types and number of input and outputs. Build input validation on the methods of your API to help users recover from run-time errors. Error messages should be clear, objective, and indicative of the problem and the context where it appeared, and propose a solution. The lack of progress and termination feedback in the ML model training stage was considered problematic. Methods or functions which complete asynchronously such as ML model training, benefit from progression and completion feedback. Provide API methods and complementary tooling (e.g., visualization libraries) for accessing the configuration and state of the ML pipelines and workflows. Use them in the examples and documentation to help users build a correct mental model from a solid pattern and prevent errors.
8. *Support the diversity of user engineering skills for ML -non-experts users*–novice developers require a strong proposition with regards to the conceptual scaffolding. This might entail creating more visual and holistic resources, which might convey more effectively the “big picture,” and creating minimal and simple examples, with a standard code styling and no optimization for readability. Experienced developers require another level of elaboration and penetrability to reach their design goals. They will value lower-level primitives for control and configuration of ML algorithms and pipelines, a wider selection of ML algorithms and more sophisticated data structures, which may yield more expressiveness in the final result. To strike this challenging balance between both ends of the spectrum of user developer-skills, it is fundamental to build an extensible and modular ML API architecture. It is also important to differentiate documentation and guides according to user development skill levels and to tailor and provide support for a more adequate learning journey.
9. *Build a thriving community and ecosystem comprising documentation, resources and infrastructure*—an active community can support new users with the on-boarding process and with troubleshooting issues. It can also give more experienced users opportunities to contribute with solutions, mentor, network, and peer-review open-source contributions and extensions to an ML API. Online fora and Q&A platforms such as StackOverflow provide the media for the community to engage and interact and keep a history of answers to issues previously raised by other users. Meetups, workshops, and hackathons can grow the community offline and strengthen its bonds.

## 6. Conclusion

This study employed a qualitative and user-centric approach to explore and better understand how ML API design may facilitate the learning, use and rapid adoption by creative software developers and music technologists. The design of an ML API can impact its adoption, the user-developers' working processes, and the client application features and interaction style. Current ML API designs and designers show awareness about the importance of adopting design principles which guide usability, learnability and accessibility. However, research focused on the human-centered design, evaluation and developer experience with ML APIs is fundamentally under-explored, in particular, of ML APIs specialized in the development of systems for creative and musical technology. This kind of user study is therefore important for how it builds upon a more nuanced connection between designers and end users of an ML API. We used an adapted version of the CDs questionnaire to explore how the design decisions and trade-offs of an API for rapid prototyping with IML relate to its usability and the developer experience.

The application of the CDs to the usability assessment of ML APIs helped uncover problems and directions of improvement, mostly related to documentation fragmentation, support for understanding intricate working details, error support and recoverability, and lack of evaluation guidance. Results also indicate that the RAPID-MIX API caters well to beginners and ML-non-expert users in general. It appears to support incremental learning approaches and to provide a low entry barrier and smooth learning curve to ML. The direct correspondence of the API to a high-level conceptual model which focuses on supervised ML learning tasks, end-to-end ML pipelines and simple data structures for datasets, appears to support effective learning, understanding and use. The structure and set of entities of this ML API support usage with minimal amount of code and context, trial-and-error exploration, easy refactoring and easy adaptation to custom user needs. This facilitates opportunistic development approaches, which are driven by design and rapid experimentation goals, and prone to happen in contexts of learning and creative and music technology development.

The CDs framework opens up interesting perspectives of analysis that support a rich and deep discussion about ML API design. However, we faced some challenges in the general application of the CDs, mostly related to communication and interpretation issues with the CDs vocabulary, and validity and reliability issues, which typically occur in questionnaire and survey methods with small samples (Adams and Cox, 2008). Other challenges relate to the difficulty to establish a scale and rate a ML API for each cognitive dimension of analysis. We also found limitations to the CDs concerning the interactions of an ML API with other artifacts, such as the text-editor or programming environment where ML API integration code is programmed, or its documentation media. Although a CD assessment cannot lead to full usability validation (Dagit et al., 2006) of an ML API, it can lead to new insights which may trigger new design iterations and thus become a useful and pragmatic resource to ML API designers.

Future work includes avenues of research which build on the CDs and quantitative methods as pragmatic methodological tools for ML API and notation designers. One avenue is to investigate a more focused and formalizable set of dimensions, which may help to analyse the use of IML and ML APIs more adequately. Another avenue of research is to explore ways to augment the cognitive dimensions framework to account more holistically for a set of interdependent artifacts—including language notations, programming interfaces, development environments, documentation and other high-level aspects which Petre (2006) has identified. Finally, we are exploring new research tools for conducting online quantitative usability studies with ML APIs which may scale to more participants and provide more generalizable outcomes.

## Data Availability Statement

The datasets generated for this study are available on request to the corresponding author.

## Ethics Statement

Ethical review and approval was not required for the study on human participants in accordance with the local legislation and institutional requirements. The patients/participants provided their written informed consent to participate in this study.

## Author Contributions

FB the primary author, designed and led the study, and contributed to the design and development of the RAPID-MIX API. MZ was the lead designer and developer of the RAPID-MIX API. MG provided high-level research guidance and contributed to all aspects of study. RF contributed to the design of the questionnaire and provided critical guidance and supervision throughout all the stages of the study. All authors contributed to the production and review of this work and to the design of the RAPID-MIX API.

### Conflict of Interest

The authors declare that the research was conducted in the absence of any commercial or financial relationships that could be construed as a potential conflict of interest.

## Footnotes

^1^Venners, B. and Eckel, B., The C# Design Process: A Conversation with Anders Hejlsberg – Part I, https://www.artima.com/intv/csdes2.html, (accessed September 15, 2019).

^2^Core ML, https://developer.apple.com/documentation/, (accessed September 15, 2019).

^3^Turi Create, https://github.com/apple/turicreate, (accessed September 15, 2019).

^4^Pytorch, https://pytorch.org/, (accessed September 15, 2019).

^5^Principal component analysis (PCA) is a statistical method which converts observations of potentially correlated variables into a set of linearly uncorrelated variables (the principal components). PCA is often applied for dimensionality reduction high-dimensional data sets.

^6^A hidden Markov model is a Markov chain for which the state is only partially observable. A Markov chain is a method for modeling complex systems using random processes and probability, sequences of possible events and interdependent states.

^7^Max, https://cycling74.com/products/max/

^8^Pure Data, https://puredata.info/

^9^https://blog.keras.io/user-experience-design-for-apis.html

^10^https://itp.nyu.edu/adjacent/issue-3/ml5-friendly-open-source-machine-learning-library-for-the-web/

^11^RAPID-MIX: Realtime Adaptive Prototyping for Industrial Design of Multimodal Interactive eXpressive technology, http://rapidmix.goldsmithsdigital.com/

^12^ROLI, https://roli.com

^13^AudioGaming, http://www.audiogaming.net/

^14^Reactable Systems, https://reactable.com

^15^LeapMotion, https://www.leapmotion.com/

^16^MYO, https://support.getmyo.com/

^17^BITalino, https://bitalino.com/en/
