# Supplementary material for: Designing and Evaluating the Usability of a Machine Learning API for Rapid Prototyping Music Technology
Source: Front Artif Intell. 2020 Apr 3;3:13. doi: 10.3389/frai.2020.00013 (PMC7861239; doi:10.3389/frai.2020.00013)
Supplement: Supplementary file 1 [file Data_Sheet_1.pdf]

APPENDIX – COGNITIVE DIMENSIONS QUESTIONNAIRE

Q1. How appropriate have you found the overall API abstraction level to be for your development needs? How would you classify the abstraction level of the types of classes and methods that you had to work with,

☐ too high level ☐ too low level ☐ just right

Why?

Q2. Did you feel that you had to understand the underlying implementation to be able to use the functionalities of the API? Why?

Q3. What previous Machine Learning (ML) knowledge made it easy to use the API?

Q4. What other areas of knowledge (other than ML) would have made it easier to use the API?

Q5. What knowledge about the API components was essential to achieve your development and implementation goals?

Q6. How did you go about learning how to use the API? (Check all that apply)

☐ I wrote a couple of lines of code to try to get something working and built up understanding from that

☐ I copied sample code provided with the API

☐ I read a high level overview of the API first; I only started writing code once I had a general idea about the architecture of the API

☐ I learned about the API components and their dependencies before starting to do anything useful related to my development goal

☐ I learned about the underlying ML architecture of the API and other conceptual information (e.g., specific algorithms, different approaches, etc) before starting to do anything useful related to my development goal.

Q7. Can you give examples of objects with unclear dependencies between them?

Q8. How would you describe your experiences in learning how to use the API? Was there

☐ too much to learn ☐ not enough to learn ☐ just right

Why?

Q9. How did the different API documentation elements supported the learning that you needed to progress with your development goals?

a) Website

☐ unsatisfactory ☐ improvement needed ☐ good ☐ very good ☐ outstanding ☐ did not use

b) Git repository and README file

☐ unsatisfactory ☐ improvement needed ☐ good ☐ very good ☐ outstanding ☐ did not use

c) Code examples

☐ unsatisfactory ☐ improvement needed ☐ good ☐ very good ☐ outstanding ☐ did not use

d) Code comments

☐ unsatisfactory ☐ improvement needed ☐ good ☐ very good ☐ outstanding ☐ did not use

Q10. What ‘context’ did you have to keep in mind in order to use the API functionalities you needed for your development goals? (Check all that apply)

☐ type(s) of ML algorithm

☐ data structure(s) for training and model input/output

☐ local scope variables

☐ global scope variables

☐ registered events

☐ API classes

☐ API methods

☐ other components

☐ app configuration settings

☐ system configuration settings

☐ database (e.g., JSON, XML, CSV, database management service)

☐ other (please describe):

Q11. How would you describe your experiences with respect to the amount of ‘context’ that you had to keep in mind to implement what you wanted?

☐ too much to learn ☐ not enough to learn ☐ just right

Why?

Q12. How would you describe your experience with respect to the overall amount of code you had to write to use the API?

Q13. Were you able to evaluate your progress in using the API when you needed to, or did it require more work than expected to evaluate progress? Why?

Q14. When you were programming with the API, were you able go about the process in any order you liked or were you forced to think ahead and make certain decisions first?

Q15. What main decisions did you need to make in advance, in order to achieve your development goals?

Q16. Was it obvious that you needed to make these decisions? Did you learn about this through trial and error?

Q17. What sort of problems did this cause in your work?

Q18. How easy is it to see or find the details of the API while you are using it? What kinds of things are more difficult to see or find? Why?

Q19. Did the API and its documentation provide enough information about the relevant machine learning specifics related to your development goal?

Q20. What information was missing, or that you had to find by referring to external sources?

Q21. How much of the underlying details of the machine learning did you have to understand in order to be able to use the API successfully? How much of those details are exposed in the API?

Q22. By what process did you understand the intricate working details of the API while you worked on your implementation?

Q23. Were you able to find enough information to understand the intricate working details of the API while you work on your programming task? If not, what are the information you think is missing?

Q24. Were you able to use the API exactly ‘as-is’ or did you feel the need to adapt the API to meet your needs? Did you (check all that apply):

☐ fork the API?

☐ build any adapter class(es) for using it?

☐ derive or extend any classes or types?

☐ override methods?

☐ hack the API?

☐ any other?

Why?

Q25. When you needed to make changes to code that you had previously written using the API, how easy was it to make these changes? Why?

Q26. If you noticed elements of the API that offered similar functionalities? Were the differences between them made clear to you? Please explain.

Q27. When reading code that uses the API, is it easy to tell what each section of code does?

☐ yes ☐ no

Q28. Can you give examples of parts that were particularly difficult to interpret?

☐ yes ☐ no

Q29. Was it easy to know which API classes and methods to use when writing code?

☐ yes ☐ no

Q30. When using the API, do the exposed classes and methods map easily to the conceptual objects that you think about manipulating?

☐ yes ☐ no

Q31. Can you give examples of any such conceptual objects that map easily? Or that do not?

Q32. When using the API, was it easy to map from your conceptual ideas and application functionalities to API code?

☐ yes ☐ no

Why?

Q33. Were there any incidents in which you used the API incorrectly?

Q34. Did the API offer any help to identify that you misused it? If any similar incidents occurred, can you please explain.

Q35. Did you eventually identify the correct way of using it?

Q36. Did the API give proper error messages in case of exceptions or error? Did you have to handle them at application level? If you had to handle them at application level, please mention the scenarios.

Q37. Did you use any of the following testing or evaluation as your application developed or after you completed it?

a) Objective evaluation with unit tests and fixtures:

☐ integration or functional test (higher level test for application areas wrapping API features)

☐ seam tests for unstable data (unit tests for validation of model tolerance between data input and outputs)

☐ training accuracy test

☐ cross validation tests of model fit

☐ precision/recall

b) Subjective evaluation with direct tests and direct observation of model behaviour:

☐ Correctness (percentage of test cases where model produces the desired result)

☐ Cost (weighted correctness; do the most important test cases produce the desired result)

☐ Decision boundary (does a classifier change label in the right place)

☐ Confidence (is the model more confident when it produces correct outputs for easy problems, and less confident when it produces outputs for hard problems, or produces incorrect outputs)

☐ Other criteria (e.g., complexity, expressive potential, unexpectedness, ease of use) — please describe:

Q38. Why did you test these things?

Q39. Did the API provided any guidance on how to test your application? Did you get guidance from elsewhere?

Q40. Do you find yourself using the API in ways that seemed unusual, or ways that the implementers might not have intended? Can you give some examples?
